# Interstitial lung disease in clinically amyopathic dermatomyositis with and without anti-MDA-5 antibody: to lump or split?

**DOI:** 10.1186/s12890-015-0154-4

**Published:** 2015-12-09

**Authors:** Satoshi Ikeda, Machiko Arita, Mitsunori Morita, Satoshi Ikeo, Akihiro Ito, Fumiaki Tokioka, Maki Noyama, Kenta Misaki, Kenji Notohara, Tadashi Ishida

**Affiliations:** Department of Respiratory Medicine, Kurashiki Central Hospital, Miwa 1-1-1, Kurashiki-city, Okayama, 710-8602 Japan; Department of Rheumatology, Kurashiki Central Hospital, Okayama, Japan; Department of Pathology, Kurashiki Central Hospital, Okayama, Japan

**Keywords:** Amyopathic dermatomyositis, Interstitial lung diseases, MDA-5 protein, human, Melanoma differentiation associated protein-5, human

## Abstract

**Background:**

Interstitial lung disease (ILD) associated with clinically amyopathic dermatomyositis (CADM-ILD) is often refractory and rapidly progressive. Although the anti-melanoma differentiation-associated gene 5 (anti-MDA-5) antibody is associated with rapidly progressive ILD (RP-ILD), differences in clinical features and prognosis of anti-MDA-5 antibody-positive and -negative CADM-ILD remain unclear.

**Methods:**

To clarify the differences in the clinical features and prognosis between anti-MDA-5 antibody-positive and -negative cases, we retrospectively reviewed the medical records of patients diagnosed with CADM-ILD with and without anti-MDA-5 antibody at Kurashiki Central Hospital from January 2005 to September 2014.

**Results:**

Anti-MDA-5 antibody was found in 10 of 16 patients (63 %). The levels of Krebs von den Lungen-6 (KL-6) and surfactant protein D (SP-D) at the first visit were significantly lower in positive patients than in negative patients, whereas the levels of aspartate aminotransferase (AST), γ-glutamyl transpeptidase (γ-GTP), and the CD4^+^/CD8^+^ ratio in the bronchoalveolar lavage (BAL) fluid were significantly higher in positive patients than negative patients. Subpleural ground-glass opacity (GGO) or irregular linear opacity was predominant in positive patients. Peribronchovascular consolidation was predominant in negative patients. Positive patients had significantly lower survival rates than negative patients, with all six fatal cases occurring in positive patients who died of refractory ILD within 92 days from the first visit despite intensive treatment.

**Conclusions:**

There are clear differences in the clinical features and prognosis of anti-MDA-5 antibody-positive and -negative CADM-ILD. Low serum KL-6 and SP-D levels, high serum AST and γ-GTP levels, high CD4^+^/CD8^+^ ratio in BAL fluid, and predominance of subpleural GGO or irregular linear opacity in HRCT may help to discriminate anti-MDA-5 antibody-positive CADM-ILD with poor prognosis.

## Background

Interstitial lung disease (ILD) is the most common internal organ manifestation that affects the prognosis of clinically amyopathic dermatomyositis (CADM), as well as polymyositis (PM) and dermatomyositis (DM). ILD associated with CADM (CADM-ILD) is often refractory and rapidly progressive [1–3], resulting in respiratory failure with a 6-month survival rate of 40.8–54.5 % [3–5]. Although no standard treatment regimen for CADM-ILD has been established, intensive treatment with the three-drug combination of corticosteroid, cyclosporine, and cyclophosphamide is recommended in the early phase even if respiratory symptoms are absent or mild [6, 7].

Among patients with CADM, anti-melanoma differentiation-associated gene 5 (MDA-5) antibodies are strongly associated with the development of rapidly progressive ILD (RP-ILD) [8–10]. As MDA-5 plays the critical role in the innate immune defense against viruses, one hypothesis is that the production of anti–MDA-5 antibodies is a secondary phenomenon during virus infection that is associated with the onset of CADM and RP-ILD. However, to our knowledge, no previous study has revealed whether the presence or absence of the anti-MDA-5 antibody affects the clinical manifestation of CADM-ILD.

In the present study, we retrospectively reviewed consecutive cases of CADM-ILD to clarify the differences in the clinical features and prognosis between anti-MDA-5 antibody-positive and -negative cases, and to determine whether we should separate CADM-ILD by the presence or absence of anti-MDA-5 antibody.

## Methods

### Patients and settings

This retrospective study was performed at Kurashiki central hospital in Kurashiki city, Okayama, Japan. The patients diagnosed with CADM-ILD at our hospital from January 2005 to September 2014 who had cryopreserved blood serum before starting treatment were enrolled in this study. Diagnoses of CADM were made by at least two pulmonologists and one rheumatologist based on the criteria of Sontheimer [11]. In addition, patients who exhibited a rash typical of DM without muscle weakness for less than 6 months and experienced fatal complications such as acute/subacute ILD were also diagnosed with CADM according to Gerami et al.’s criteria [12]. No exclusion criteria were specified. The Ethics Committee of Kurashiki Central Hospital approved the study protocol. The Ethics Committee approved the waiver of each patient’s consent because it was a retrospective study and high anonymity was secured.

### Identification of myositis-specific antibodies

Measurement of myositis-specific antibodies was carried out by using cryopreserved blood serum before starting treatment. Serum anti-MDA-5 antibody was measured by immunoprecipitation using ^35^S-labeled HeLa cell extract (Perkin Elmer, Waltham, MA, USA), which was confirmed by enzyme-linked immunosorbent assay (SRL, Tokyo, Japan). Other myositis-specific antibodies, including anti-aminoacyl transfer RNA synthetase (anti-ARS) antibodies, were measured using the Myositis Profile Euroline antibody test system (EUROIMMUN, Lubeck, Germany), which was confirmed by RNA immunoprecipitation (Bio-Rad Laboratories, Hercules, CA, USA).

### Clinical and laboratory findings

Clinical data and laboratory data used in the present study were retrieved from patient medical records and included gender, age, smoking history, length of time from onset of symptoms to first visit, department of the first visit, symptoms and physical examination, laboratory data, and results of bronchoalveolar lavage (BAL) and pulmonary function tests. BAL was routinely performed under local anesthesia before starting treatment. The bronchoscope was wedged into the segmental bronchi. Only samples with recovery >25 % were used. With regards to pulmonary function test, forced vital capacity and diffusing capacity for carbon monoxide were measured using same machine and same method in all patients. Values were expressed as a percentage of the predicted value.

### Radiological findings

All patients underwent high-resolution computed tomography (HRCT) at the time of diagnosis, and HRCT findings were reviewed and interpreted by board-certified pulmonologists and radiologists. Images were assessed for the dominant craniocaudal/axial distribution and the dominant shadow [consolidation, ground-glass opacity (GGO), reticulation, or irregular linear opacity]. The presence of traction bronchiectasis, cyst, subpleural curve linear shadow, thickening of the perilymphatic interstitium, emphysema, and loss in lung volume were also assessed. HRCT protocol and machine was the same for all included patients at peak tube voltage of 120 kVp and approximately ≤240 mAs using an automatic exposure control system (Toshiba Medical Systems, Tochigi, Japan).

### Statistical analysis

Categorical data are presented as numbers (percentages), while continuous data are presented as medians (interquartile ranges). Fisher’s exact test was used to compare categorical data, and the Mann–Whitney *U* test was used to compare continuous data. Cumulative survival probabilities were estimated using the Kaplan-Meier method. The log-rank test was used to compare survival among patient groups. A *p* value of <0.05 was considered statistically significant.

## Results

### Characteristics

From January 2005 to September 2014, we encountered 18 cases of newly diagnosed ILD associated with CADM. Anti-MDA-5 antibody was measured in 16 cases who had cryopreserved blood serum before starting treatment. Anti-MDA-5 antibody was present in 10 patients (positive group) and absent in 6 patients (negative group). Patients’ characteristics are summarized in Table 1. The median length of time from onset to first visit was shorter in the positive group (15.5 vs. 51.0 days, respectively), although the difference did not reach statistical significance (*p* = 0.157). No significant differences were observed in gender, age, smoking history, or symptoms and signs at the time of diagnosis between the two groups.

**Table 1:** Summary of clinical characteristics

	MDA-5 positive	MDA-5 negative	*p* value
	(*n* = 10)	(*n* = 6)	
Gender (male/female)	6/4	2/4	0.608
Age	63.0 (58.0–64.8)	71.5 (64.8–73.8)	0.0913
Smoking history	5 (50 %)	2 (33 %)	0.633
Days from onset to first visit	15.5 (11.0–29.8)	51.0 (39.5–77.5)	0.157
Department of the first visit			
Respiratory medicine	6 (60 %)	1 (17 %)	–
Rheumatology	2 (20 %)	5 (83 %)	–
Dermatology	2 (20 %)	0	–
Symptoms and signs at the time of diagnosis			
Gottron’s sign/papule	7 (70 %)	5 (83 %)	1.00
Heliotrope eruption	7 (70 %)	1 (17 %)	0.119
V/shawl neck sign	5 (50 %)	2 (33 %)	0.633
Mechanic’s hands	2 (20 %)	2 (33 %)	0.604
Palmar erythema	2 (20 %)	1 (17 %)	1.00
Myalgia	3 (30 %)	0	0.250
Dry cough	5 (50 %)	4 (67 %)	0.633
Dyspnea on exertion	5 (50 %)	5 (83 %)	0.307
Fever	9 (90 %)	3 (50 %)	0.118

Categorical data are presented as numbers (percentages) and were analyzed using Fisher’s exact test. Continuous data are presented as medians (interquartile ranges) and were analyzed using the Mann–Whitney *U* test. A *p* value of <0.05 was considered statistically significant

*Abbreviation*: MDA-5, anti-melanoma differentiation-associated gene 5

### Laboratory data

**Fig. 1:**
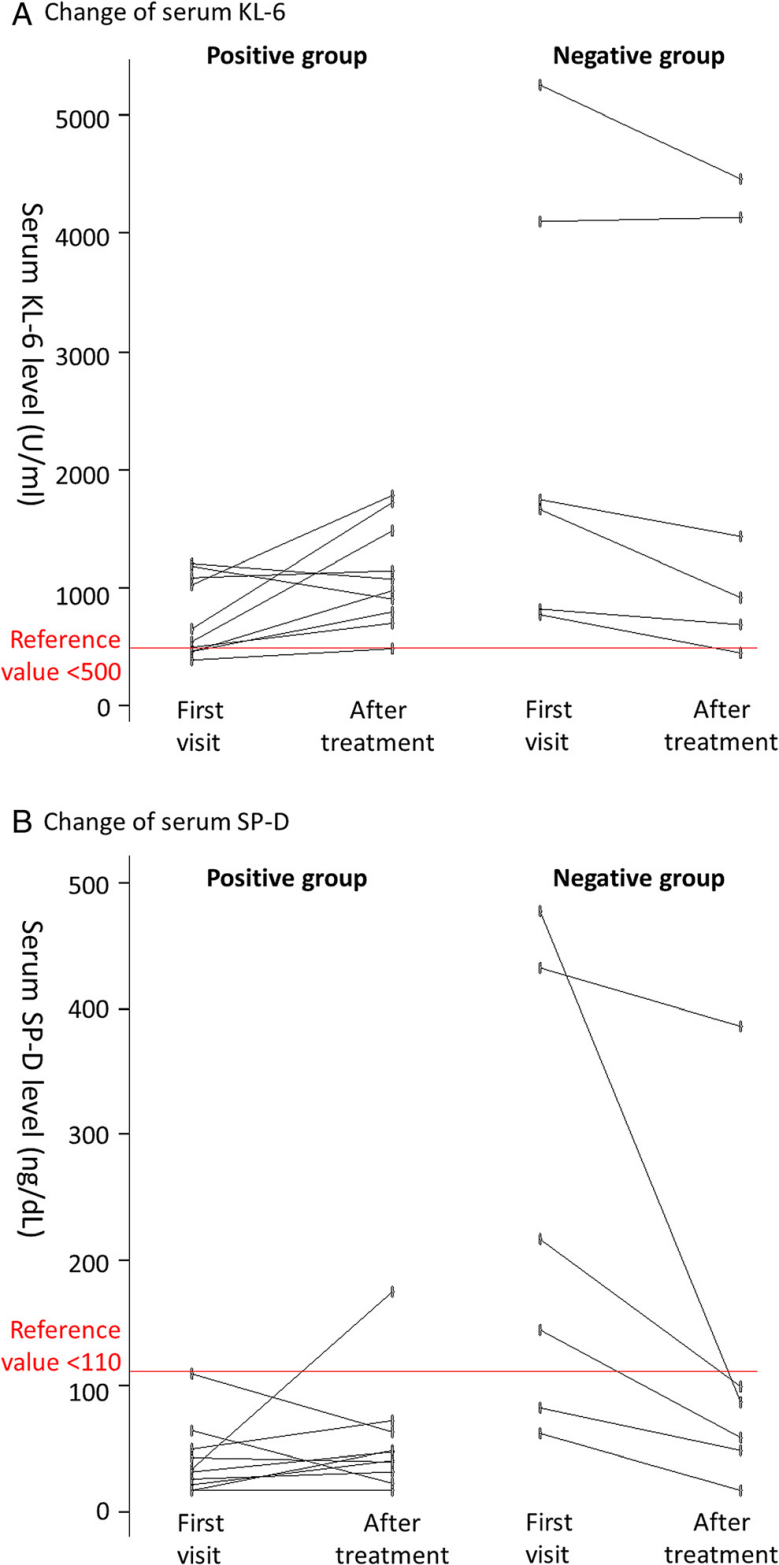
Chronological changes in serum KL-6 and SP-D. **a** Serum KL-6 levels at first visit and after 1–4 weeks’ treatment initiation in each patient of the anti-MDA-5 antibody positive (left) and negative groups (right). **b** Serum SP-D levels at first visit and after 1–4 weeks’ treatment initiation in each patient of the anti-MDA-5 antibody positive (left) and negative groups (right). Abbreviations: KL-6, Krebs von den Lungen-6; SP-D, surfactant protein D; MDA-5, anti-melanoma differentiation-associated gene 5

Laboratory data are summarized in Table 2. Aspartate aminotransferase (AST) and γ-glutamyl transpeptidase (γ-GTP) levels were significantly higher (*p* = 0.0225 and 0.00225, respectively) in the positive group when compared to the negative group, whereas serum levels of Krebs von den Lungen-6 (KL-6) and surfactant protein D (SP-D) at the first visit were significantly lower (*p* = 0.0160 and 0.00402, respectively) in the positive group than in the negative group. In the positive group, serum KL-6 gradually increased, whereas serum SP-D remained consistently low 1–4 weeks after treatment initiation (Fig. 1). In the negative group, one patient each was positive for anti-Jo1 antibody, anti-PL-12 antibody, anti-OJ antibody, and anti-PM/Scl-100 antibody. Regarding BAL fluid analysis, the CD4^+^/CD8^+^ ratio was significantly higher in the positive group than in the negative group (*p* = 0.0167).

**Table 2:** Summary of the results of laboratory testing, bronchoalveolar lavage, and pulmonary function test

	MDA-5 positive	MDA-5 negative	*p* value
	(*n* = 10)	(*n* = 6)	
PaO_2_/FiO_2_ ratio	296 (274–359)	343 (332–357)	0.689
Laboratory data			
White blood cell count (/μL)	5600 (4825–6300)	8150 (5775–10150)	0.573
Aspartate aminotransferase (IU/L)	55.5 (40.5–92.3)	28.5 (23.5–33.5)	0.0225
Alanine aminotransferase (IU/L)	27.0 (17.0–105)	17.0 (16.3–19.3)	0.252
γ-glutamyl transpeptidase (IU/L)	40.5 (23.0–60.5)	15.5 (14.0–22.3)	0.00225
Creatine phosphokinase (IU/L)	184 (90.3–467)	108 (85.8–268)	0.635
Aldolase (U/L)	6.15 (3.58–8.45)	7.50 (4.45–8.45)	0.713
C-reactive protein (mg/dL)	1.28 (0.90–2.94)	0.62 (0.36–3.53)	0.428
Lactate dehydrogenase (IU/L)	368 (302–390)	297 (259–331)	0.0925
Krebs von den Lungen-6 (U/mL)	600 (471–1072)	1700 (1027–3511)	0.0160
Surfactant protein D (ng/dL)	32.5 (22.8–47.6)	181 (98.1–378)	0.00402
Anti-ARS antibody	0	3 (50 %)	
Anti Jo-1	0	1 (17 %)	
Anti PL-12	0	1 (17 %)	
Anti 0 J	0	1 (17 %)	
Anti-PM/Scl-100 antibody	0	1 (17 %)	
Bronchoalveolar lavage			
Total cell count	300 (250–400)	500 (450–700)	0.131
Neutrophil (%)	3.00 (2.50–6.50)	11.0 (8.50–51.5)	0.0855
Eosinophil (%)	1.00 (0.00–1.00)	4.00 (2.00–5.50)	0.284
Lymphocyte (%)	30.0 (24.5–37.0)	41.0 (22.0–42.0)	0.819
Macrophage (%)	60.0 (59.5–70.5)	41.0 (23.0–44.0)	0.0396
CD4+/CD8+ ratio	1.68 (1.13–3.62)	0.420 (0.340–0.490)	0.0167
Lung function test			
% Forced vital capacity	79.8 (67.4–90.4)	72.9 (53.4–93.6)	1.00
% Diffusing capacity for carbon monoxide	56.6 (50.2–63.7)	56.9 (54.7–63.0)	0.730

Categorical data are presented as numbers (percentages) and were analyzed using Fisher’s exact test. Continuous data are presented as medians (interquartile ranges) and were analyzed using the Mann–Whitney *U* test. A *p* value of <0.05 was considered statistically significant

*Abbreviation*: MDA-5, anti-melanoma differentiation-associated gene 5

### HRCT findings

**Fig. 2:**
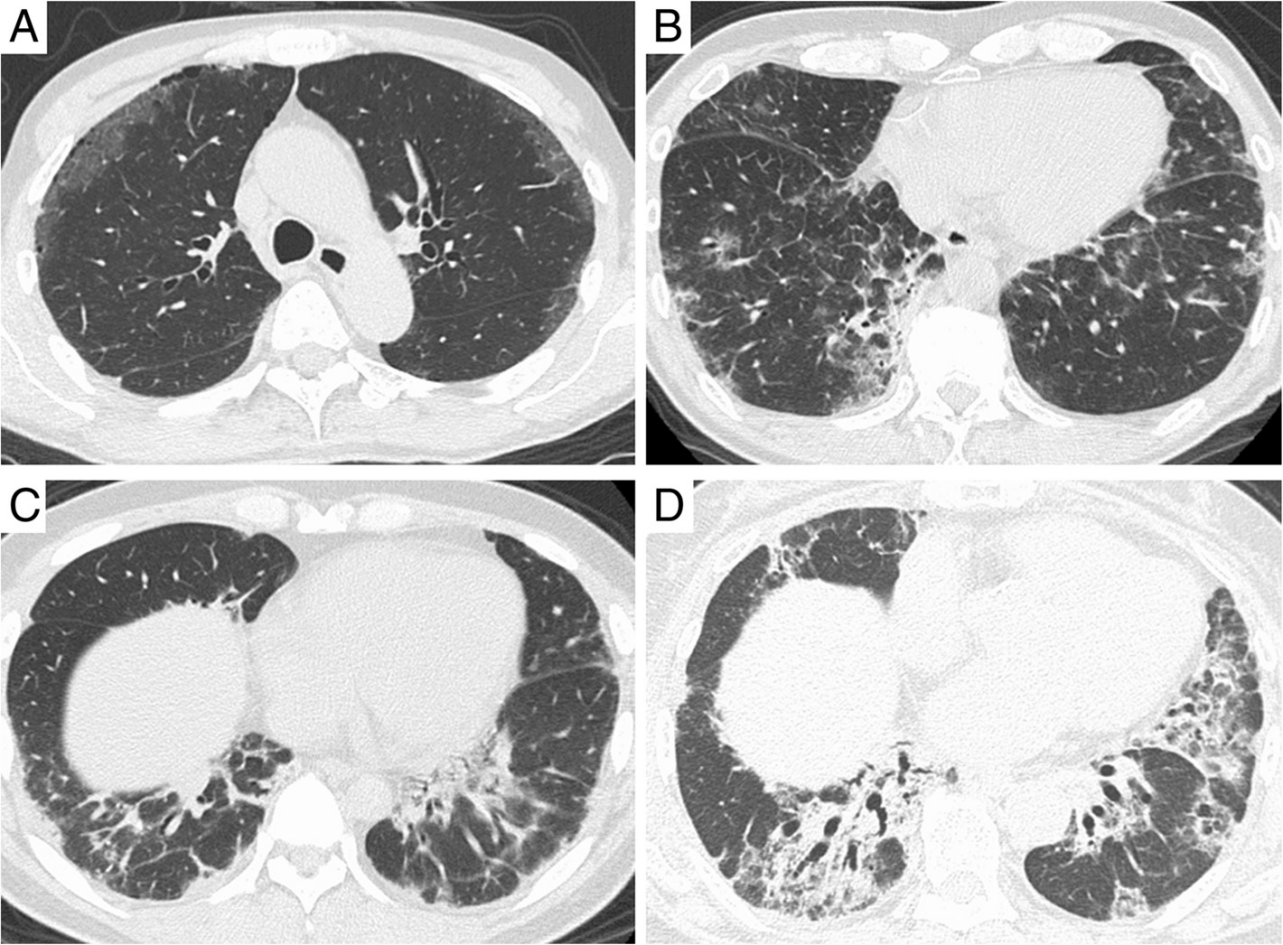
High-resolution computed tomography findings. Representative photographs of HRCT scans are presented. **a** and **b** initial HRCT scans of fatal cases positive for anti-MDA-5 antibody showing subpleural GGO. (C) HRCT scans of an anti-MDA-5 antibody-positive patient who survived, showing subpleural irregular linear opacity. (D) HRCT scans of an anti-MDA-5 antibody-negative patient showing peribronchovascular consolidation. Abbreviations: GGO, ground-glass opacity; HRCT, high-resolution computed tomography; MDA-5, anti-melanoma differentiation-associated gene 5

HRCT findings are shown in Table 3 and Fig. 2. In both groups, there was a predominance of shadows in the lower lobe. Subpleural distribution was predominant in the positive group, whereas peribronchovascular distribution was predominant in the negative group. In the positive group, GGO (50 %) was the most commonly observed shadow followed by irregular linear opacity (30 %). Conversely, in the negative group, the most common shadow was consolidation with a significantly higher incidence than in the positive group (*p* = 0.00762). While HRCT findings in the positive and negative group often appeared to be mild, most were associated with loss of lung volume (90 and 83 %, respectively) or traction bronchiectasis (50 and 67 %, respectively).

**Table 3:** Comparison of HRCT findings between patients with and without anti-CADM-antibody

	MDA-5 positive	MDA-5 negative	*p* value
	(*n* = 10)	(*n* = 6)	
Distribution			
Upper lobe dominant	1 (10 %)	0	1.00
Lower lobe dominant	9 (90 %)	6 (100 %)	1.00
Peribronchovascular	2 (20 %)	4 (67 %)	0.118
Subpleural	8 (80 %)	2 (33 %)	0.118
Main findings			
Consolidation	1 (10 %)	5 (83 %)	0.00762
Ground glass opacity	5 (50 %)	0	0.0934
Reticulation	1 (10 %)	0	1.00
Irregular linear opacity	3 (30 %)	1 (17 %)	1.00
Additional findings			
Traction bronchiectasis	5 (50 %)	4 (67 %)	0.633
Cyst	0	1 (17 %)	0.375
Subpleural curve linear shadow	4 (40 %)	3 (50 %)	1.00
Thickening of interlobular septa	2 (20 %)	2 (33 %)	0.604
Emphysema	1 (10 %)	1 (17 %)	1.00
Volume loss	9 (90 %)	5 (83 %)	1.00

Categorical data are presented as numbers (percentages) and were analyzed using Fisher’s exact test

*Abbreviations*: *CADM* clinically amyopathic dermatomyositis, *HRCT* high-resolution computed tomography, MDA-5, anti-melanoma differentiation-associated gene 5

### Treatments and outcomes

Three-drug combination therapy with corticosteroid, cyclosporine, and cyclophosphamide was the most common initial treatment in both groups (78 and 83 %, respectively). The median follow-up period over both groups was 689 days (the data cutoff date was November 9, 2014). In the present study, six deaths were observed during the follow-up period; all six cases were positive for anti-MDA-5 antibody and died from refractory ILD within 92 days from the first visit (median, 30.0 days). Five of these six cases received intensive initial treatment with corticosteroid, cyclosporine, and cyclophosphamide. The one remaining case was refractory to the initial treatment with corticosteroid monotherapy, and thus subsequently received cyclosporine and cyclophosphamide.

**Fig. 3:**
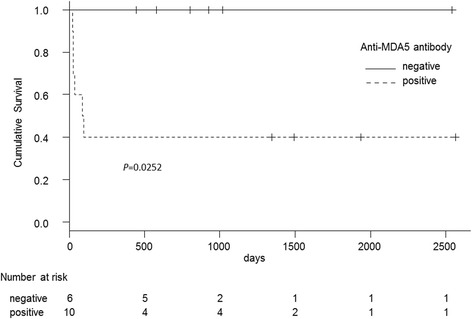
Comparison of survival curves with and without anti-MDA-5 antibody. Cumulative survival probabilities were estimated using the Kaplan–Meier method. The log-rank test was used to compare survival among patient groups. A *p* value of <0.05 was considered statistically significant. Abbreviation: MDA-5, anti-melanoma differentiation-associated gene 5

A comparison of survival curves is shown in Fig. 3. The anti-MDA-5 antibody-positive group had significantly lower survival rates than the negative group (*p* = 0.0252).

### Comparison between survivors and non-survivors with anti-MDA-5 antibody

A comparison between the survivors and non-survivors in the positive group is shown in Table 4. The length of time from onset to the first visit was shorter among the non-survivors than the survivors, although this difference did not reach statistical significance (*p* = 0.0666). The length of time from onset to treatment initiation was significantly shorter in non-survivors than in survivors (*p* = 0.0381). As for the HRCT findings, the incidence of GGO was significantly higher among non-survivors than survivors (*p* = 0.0480). No significant differences were observed in terms of gender, age, smoking history, PaO_2_/FiO_2_ ratio, or BAL fluid analysis results between the two groups.

**Table 4:** Comparisons of characteristics and examination findings between anti-MDA-5-positive survivors and non-survivors

	Survivors	Non-survivors	*p* value
	(*n* = 4)	(*n* = 6)	
Gender (male/female)	2/2	4/2	1.00
Age	58.5 (47.8–64.3)	63.0 (59.3–64.5)	0.519
Time			
from onset to first visit	29.5 (26.0–50.3)	11.0 (11.0–13.3)	0.0666
from onset to treatment initiation	68.5 (31.5–107)	16.0 (12.8–23.0)	0.0381
Symptoms and signs			
Gottron’s sign	2 (50 %)	5 (83 %)	0.500
Heliotrope eruption	2 (50 %)	5 (83 %)	0.500
V/shawl neck sign	1 (25 %)	4 (67 %)	0.524
Mechanic’s hands	0	2 (33 %)	0.467
Palmar erythema	0	2 (33 %)	0.467
Myalgia	2 (50 %)	1 (17 %)	0.500
Cough	4 (100 %)	1 (17 %)	0.0480
Dyspnea on exertion	3 (75 %)	2 (33 %)	0.524
Fever	3 (75 %)	6 (100 %)	0.200
Laboratory data			
Aspartate aminotransferase	55.5 (36.8–76.8)	55.5 (41.0–110)	0.762
γ-glutamyl transpeptidase	53.5 (43.5–57.5)	28.0 (23.0–110)	0.914
Krebs von den Lungen-6	1135 (930–1190)	524 (470–626)	0.114
Surfactant protein D	45.3 (23.7–75.8)	32.5 (24.2–40.9)	0.669
Bronchoalveolar lavage			
CD4+/CD8+ ratio	3.52 (2.12–4.04)	1.47 (1.20–2.19)	0.857
HRCT findings			
Consolidation	1 (25 %)	0	0.400
Ground glass opacity	0	5 (83 %)	0.0480
Reticulation	1 (25 %)	0	1.00
Irregular linear opacity	2 (50 %)	1 (17 %)	0.500

Categorical data are presented as numbers (percentages) and were analyzed using Fisher’s exact test. Continuous data are presented as medians (interquartile ranges) and were analyzed using the Mann–Whitney *U* test. A *p* value of <0.05 was considered statistically significant

*Abbreviations*: *HRCT* high-resolution computed tomography, MDA-5, anti-melanoma differentiation-associated gene 5

## Discussion

Previous studies, mainly from Asia, have demonstrated that CADM-ILD often runs an aggressive course [3–5]. On the contrary, Cottin et al. reported good treatment response and favorable prognosis of CADM-ILD in France [13]. These results suggest that CADM-ILD includes a heterogeneous disease population. The present study demonstrated the four following important clinical observations. First, serum KL-6 and SP-D at the first visit were significantly lower in the positive group than in the negative group. Second, serum AST, γ- GTP, and CD4^+^/CD8^+^ ratio in the BAL fluid were significantly higher in the positive group than in the negative group. Third, radiological findings were quite different between the two groups. Fourth, anti-MDA-5 antibody-positive cases had significantly lower survival rates than anti-MDA-5 antibody-negative cases. These clinical differences imply that anti-MDA-5 antibody-positive and -negative CADM-ILD should be regarded as separate entities.

The biomarkers ILD, KL-6, and SP-D are reportedly useful for assessing the prognosis of ILD in PM and DM [14–16]. However, the usefulness of KL-6 and SP-D in CADM-ILD has not been fully investigated in previous research. In the present study, serum levels of KL-6 at the first visit were lower in the positive group than in the negative group but gradually increased in most of the cases during the observation period. Although the cause is not clear, the two following reports may provide clues for resolving this problem. First, Otsuka et al. reported that in the early stage of acute exacerbation of idiopathic pulmonary fibrosis, the elevation in serum KL-6 mostly occurred after the manifestation of symptoms and deterioration of HRCT findings and the other biomarkers of ILD [17]. Second, Sakamoto et al. reported that the serum levels of KL-6 are significantly correlated with the extent of traction bronchiectasis observed in HRCT, which is an indicator of the pathologic grade of fibrosis [18]. As anti-MDA-5 antibody-positive cases tend to progress more rapidly compared with anti-MDA-5 antibody-negative cases, serum KL-6 levels may not yet have increased in anti-MDA-5 antibody-positive cases at the first medical examination. However, the higher values of KL-6 in the negative group might be somewhat affected by 2 subjects who have especially high KL-6 at first visit, thus we must be careful in interpreting the results. On the other hand, serum SP-D levels in the positive group were significantly lower than in the negative group and remained consistently low during the course of treatment. In the negative group, serum SP-D levels decreased after anti-inflammatory treatment in most of the cases. Several studies have reported that serum SP-D concentrations are correlated with the extent of alveolitis (most commonly reflected by increased cellularity in the alveolar interstitium), but not with the progression of fibrosis [19]. Thus, low serum SP-D levels in the positive group may reflect poor cellularity and progressive fibrotic change, whereas high serum SP-D levels in the negative group may reflect relatively abundant cellularity.

As a background to the differences in the KL-6 and SP-D levels, we believe that immune reactions may differ in the lung between the two groups. The following two results support this supposition. 1) Serum levels of AST and γ-GTP were significantly higher in the positive group than in the negative group. Elevation of serum hepatobiliary enzymes was reported to be correlated with ILD in anti-MDA-5 antibody positive DM and CADM patients [20–22]. It was supposed that anti-MDA-5 antibody is associated with alveolar macrophage activation, thereby causing injury not only to the skin and lung, but also to the liver. 2) Furthermore, the CD4^+^/CD8^+^ ratio in the BAL fluid was significantly higher in the positive group than in the negative group. No previous reports have compared the results of BAL analysis between anti-MDA-5 antibody-positive and anti-MDA-5 antibody-negative CADM-ILD patients. Although it remains controversial whether the BAL lymphocyte subset is useful for clinical diagnosis of ILD, Suda et al. reported a higher CD4^+^/CD8^+^ ratio in patients with acute/subacute CADM-ILD than chronic CADM-ILD [23]. Mukae et al. also showed a higher CD4^+^/CD8^+^ ratio in the BAL fluid of CADM patients than in those with classic DM [5]. In addition, Ito et al. found a higher CD4^+^/CD8^+^ ratio in the BAL fluid of DM patients with RP-ILD than DM patients with chronic ILD [24]. These results provide further confirmation of our data.

As well as laboratory data, radiological findings were quite different between the two groups. In the present study, the most common HRCT pattern in the positive group was lower subpleural GGO followed by subpleural irregular linear opacity. Although no previous reports have clarified HRCT findings of anti-MDA-5 antibody-positive CADM-ILD, Tanizawa et al. reported that the most common HRCT pattern in anti-MDA-5 antibody-positive DM-ILD is lower GGO/consolidation (50 %) followed by random GGO (33 %) [22]. Irregular linear opacities were also reported to be typical in CADM-ILD [13, 23, 25]. Intriguingly, the prevalence of GGO was significantly higher in non-survivors among anti-MDA-5 antibody-positive cases in the present study. Moreover, in one anti-MDA-5 antibody-positive case with subpleural GGO (as shown in Fig. 2a), autopsy revealed diffuse alveolar septal thickening due to organizing fibrosis, airspace organization, and hyaline membranes, suggesting a diffuse alveolar damage (DAD) pattern. Although no reports have evaluated the histopathological findings of treatment-naïve CADM-ILD with anti-MDA-5 antibody, the pathological findings of autopsied lung in CADM-ILD with anti-MDA-5 antibody mostly revealed a DAD pattern [26–28]. Therefore, GGO in HRCT of anti-MDA-5 antibody-positive CADM-ILD may reflect the histopathology of DAD. On the contrary, peribronchovascular consolidation, which is suggestive of nonspecific interstitial pneumonia or organizing pneumonia, was most frequently observed in the negative group. Such CT patterns are generally observed in ILD with classic DM; in fact, anti-ARS antibody was detected in 50 % of the negative cases.

These theories on the correlation between HRCT findings and pathological findings are supported by the differences in treatment response and prognosis between the two groups. The anti-MDA-5 antibody-positive cases had significantly lower survival rates than anti-MDA-5 antibody-negative cases; all six fatal cases were positive for anti-MDA-5 antibody and died from refractory ILD despite intensive initial treatment, whereas favorable treatment response and prognosis were observed in the negative group.

As described above, there are clear differences in the clinical features and prognosis between anti-MDA-5 antibody-positive and anti-MDA-5 antibody-negative CADM-ILD; thus, it would be appropriate to separate CADM-ILD by the presence or absence of anti-MDA-5 antibody. However, only a few medical facilities can measure anti-MDA-5 antibody levels, and it takes time to obtain test results; thus, the anti-MDA-5 antibody is not very helpful for deciding on a treatment policy and forecasting prognosis. Low levels of serum KL-6 and SP-D, high levels of serum AST, γ-GTP, and CD4^+^/CD8^+^ ratio in BAL fluid, and the predominance of subpleural GGO or irregular linear opacity in HRCT may provide clues for discriminating anti-MDA-5 antibody-positive CADM-ILD with poor prognosis. On the other hand, the clinical features of anti-MDA-5 antibody-negative CADM-ILD appear rather similar to those of classical DM-ILD, and, thus, should probably not be considered as a distinct clinical entity from classic DM; continuous monitoring of muscle and other clinical symptoms is required.

A limitation of the present study is the retrospective single-center study design. The number of included patients was small, and the distribution of patients may have been skewed. Another important limitation to this study is the risk of multiple comparison testing. Because of the high number of statistical tests in spite of small number of the patients, some of these will yield a *p*-value of <0.05 by chance alone. As for ILD associated with CADM, there are regional and racial differences in the prevalence of ILD and the ratio of RP-ILD to the total population of CADM-ILD [1–4]. Additionally, the prevalence of anti-MDA-5 antibody is reportedly higher in Eastern Asia than in Europe or the US, although no previous studies have made direct comparisons [20, 21, 24, 29–31].

## Conclusions

Anti-MDA-5 antibody-positive and anti-MDA-5 antibody-negative CADM-ILD have clearly different clinico-radiological features and prognosis, and, thus, it would be appropriate to separate CADM-ILD by the presence or absence of anti-MDA-5 antibody. Further investigation with more cases is required to identify factors associated with poor prognosis among anti-MDA-5 antibody-positive CADM-ILD cases.

## Abbreviations

ILD: Interstitial lung disease
CADM: Clinically amyopathic dermatomyositis
MDA-5: Melanoma differentiation-associated gene 5
RP-ILD: Rapidly progressive ILD
KL-6: Krebs von den Lungen-6
SP-D: Surfactant protein D
AST: Aspartate aminotransferase
γ-GTP: γ-glutamyl transpeptidase
BAL: Bronchoalveolar lavage
GGO: Ground-glass opacity
HE: Hematoxylin and eosin
HRCT: High-resolution computed tomography
